# Fraction-Dependent Polyphenolic Profile and Biological Activities of *Juniperus communis* Pseudo-Fruit Extracts: Antioxidant, Antimicrobial and Selective Antimelanoma Effects

**DOI:** 10.3390/antiox15060738

**Published:** 2026-06-10

**Authors:** Alina Arabela Jojić, Nicoleta Anamaria Pașcalau, Aureliana Gabriela Antal, Diana Uţu, Delia Muntean, Laurian Vlase, Ana-Maria Vlase, Elena-Alina Moacă, Graţiana Ruse, Larisa Bihoi-Rădoi, Codruța Şoica, Diana-Simona Tchiakpe-Antal

**Affiliations:** 1 Department of Pharmaceutical Botany, Faculty of Pharmacy, “Victor Babes” University of Medicine and Pharmacy Timisoara, 2nd Eftimie Murgu Square, 300041 Timisoara, Romania; alina.jojic@umft.ro (A.A.J.); gratiana.ruse@umft.ro (G.R.); diana.antal@umft.ro (D.-S.T.-A.); 2 Department of Psycho-Neuroscience and Recovery, Faculty of Medicine and Pharmacy, University of Oradea, 410087 Oradea, Romania; 3 Research Centre for Experimental Pharmacology and Drug Design (X-Pharm Design), “Victor Babes” University of Medicine and Pharmacy Timisoara, 2nd Eftimie Murgu Square, 300041 Timisoara, Romania; gabriela.antal@umft.ro (A.G.A.); codrutasoica@umft.ro (C.Ş.); 4 Department of Pharmacology-Pharmacotherapy, Faculty of Pharmacy, “Victor Babes” University of Medicine and Pharmacy Timisoara, 2nd Eftimie Murgu Square, 300041 Timisoara, Romania; diana.utu@umft.ro; 5 Discipline of Microbiology, Faculty of Medicine, “Victor Babes” University of Medicine and Pharmacy Timisoara, 2nd Eftimie Murgu Square, 300041 Timisoara, Romania; muntean.delia@umft.ro; 6 Pharmaceutical Technology and Biopharmacy Department, Faculty of Pharmacy, “Iuliu Hațieganu” University of Medicine and Pharmacy, 8 Victor Babeș Street, 400012 Cluj-Napoca, Romania; laurian.vlase@umfcluj.ro (L.V.); gheldiu.ana@umfcluj.ro (A.-M.V.); 7 Department of Toxicology and Drug Industry, Faculty of Pharmacy, “Victor Babes” University of Medicine and Pharmacy Timisoara, 2nd Eftimie Murgu Square, 300041 Timisoara, Romania; alina.moaca@umft.ro; 8 Faculty of Medicine, “Victor Babes” University of Medicine and Pharmacy Timisoara, 2nd Eftimie Murgu Square, 300041 Timisoara, Romania; larisa.bihoi-radoi@rezident.umft.ro

**Keywords:** *Juniperus communis*, ethyl acetate-soluble fraction, *n*-butanol-soluble fraction, antioxidant activity, A375 cells, polyphenols

## Abstract

The cone berries of *Juniperus communis* L. are rich in bioactive compounds, but biological properties of extracts are strongly influenced by the solvents used to obtain them. Therefore, this study aimed to evaluate the effect of solvent fractionation on the targeted polyphenolic profile and associated antioxidant, antimicrobial, and anticancer activities of pseudo-fruit extracts. The crude ethanolic extract was subjected to liquid–liquid partitioning to obtain ethyl acetate and *n*-butanol-soluble fractions, which were characterized by HPLC–MS and FTIR, while total polyphenol content was determined using the Folin–Ciocâlteu method and biological activities were assessed through DPPH, antimicrobial assays, and in vitro cytotoxicity on A375 melanoma and HaCaT keratinocyte cell lines. The ethyl acetate-soluble fraction showed the highest polyphenol content (361.08 ± 17.72 mg chlorogenic acid equivalents/g extract) and was enriched in hyperoside, whereas the *n*-butanol-soluble fraction contained lower phenolic levels and higher rutoside content; both fractions exhibited antioxidant activity correlated with phenolic content and weak-to-moderate antimicrobial activity, particularly against *Streptococcus pyogenes*. Cytotoxicity assays revealed a dose-dependent antiproliferative effect, with the ethyl acetate fraction displaying higher activity and greater selectivity toward melanoma cells, confirmed by apoptosis-related morphological changes. These findings demonstrate that solvent polarity plays a critical role in enriching bioactive phytochemicals and support the potential of *J. communis* fractions as sources of antioxidant and selective anticancer compounds.

## 1. Introduction

The increasing demand for naturally derived therapeutic agents has stimulated extensive research into medicinal plants as important sources of structurally diverse bioactive metabolites. Owing to their long history of use in traditional medicine and their wide range of biological properties, plant-derived preparations continue to attract considerable scientific interest. Numerous phytochemicals isolated from medicinal plants have demonstrated antioxidant, antimicrobial, anti-inflammatory, and anticancer activities, highlighting their potential value for pharmaceutical development [1]. Despite the remarkable progress achieved in synthetic drug discovery, medicinal plants remain an essential component of healthcare systems worldwide. In many developing regions, traditional herbal remedies continue to represent the primary therapeutic option for a substantial proportion of the population, reflecting both their accessibility and long-standing cultural acceptance [2].

Historically, the use of medicinal plants was largely limited to crude preparations such as infusions, decoctions, and macerates. However, advances in phytochemical research and analytical technologies have enabled the isolation, characterization, and biological evaluation of individual plant-derived constituents, considerably expanding their therapeutic relevance [3].

In recent decades, the connection between traditional botanical therapies and modern pharmacology has strengthened, as plant-derived bioactive compounds have demonstrated notable therapeutic benefits across a range of diseases [4]. Interest in plant-derived bioactive compounds has grown considerably in recent years, partly as a result of concerns regarding the limitations and potential adverse reactions associated with certain conventional drugs, as well as the ongoing pursuit of novel therapeutic options with improved safety profiles [5]. Furthermore, the growing emphasis on sustainable and integrative healthcare approaches has further highlighted the importance of traditional medical knowledge, positioning the study of plant-based remedies as both historically meaningful and highly relevant to the development of innovative therapeutic strategies [6,7].

*Juniperus communis* L. is an evergreen conifer belonging to the Cupressaceae family and represents one of the most widely distributed species of the genus *Juniperus*. Owing to its remarkable ecological adaptability, the species occurs across a broad geographical range spanning both the Eastern and Western Hemispheres and can thrive under diverse environmental conditions, including forested habitats, mountainous regions, and coastal ecosystems [8,9]. The species displays remarkable ecological adaptability, growing in dry pinewoods, mixed forests, river slopes, and maritime conditions [8].

Depending on environmental factors and developmental stage, *J. communis* may occur as a low-growing shrub or as a small tree. The species is characterized by needle-shaped leaves arranged in whorls of three and by dioecious reproductive structures, with male and female cones developing on separate plants [10,11].

Following maturation, the female cones develop into fleshy berry-like structures commonly referred to as pseudo-fruits, which require approximately 18 months to reach full maturity [11,12,13].

Traditionally, juniper cone berries held an important place in folk medicine, where they have been employed for managing urinary tract infections, respiratory ailments, and digestive disorders [14]. Beyond their medicinal uses, the cone berries are widely appreciated in gastronomy as flavoring agents, particularly in the manufacture of alcoholic beverages like gin and in meat dishes [15,16]. Their distinctive aromatic profile rich in diverse terpenes imparts a fresh, woody, and subtly fruity scent, contributing to their popularity not only in gastronomy but also in aromatherapy and perfumery [10,12].

Phytochemical investigations have revealed a complex chemical composition in *J. communis* pseudo-fruits. The identified constituents comprise a broad spectrum of primary and secondary metabolites, including terpenoids, phenolic compounds, flavonoids, lignans, and various organic acids, together with carbohydrates, proteins, and waxy substances [17,18,19].

The volatile fraction, which constitutes juniper essential oil, has been extensively investigated [20]. This fraction is mainly composed of monoterpene hydrocarbons such as α-pinene, β-pinene, myrcene, sabinene, and limonene, as well as oxygenated monoterpenes including terpinen-4-ol and borneol. Sesquiterpenes such as germacrene and β-caryophyllene are also present [8,20,21]. The quantity and profile of volatile constituents in juniper cone berries show considerable variability, with reported contents ranging from less than 0.5% to more than 3.5% [10].

Previous reviews have comprehensively summarized the phytochemical diversity of *Juniperus* species, documenting the presence of phenolic acids, flavonoids, coumarins, anthocyanins, flavan-3-ols, proanthocyanidins, carotenoids, and numerous other bioactive metabolites [22,23,24,25]. In addition, several studies have highlighted the pharmacological relevance of individual phenolic compounds identified in *J. communis*, including protocatechuic acid and related derivatives [18,26].

To date, phytochemicals from juniper have been associated with several pharmacological properties, including antimicrobial [27,28,29], antifungal [30,31], analgesic [32], antioxidant [33], anti-inflammatory [34,35,36], antidiabetic [37], diuretic [10], and hepatoprotective activities [33]. Additionally, protective effects on organs such as the liver and kidneys, as well as potential anticancer and cytotoxic properties, have been reported [10,24,33,38,39].

Previous studies demonstrated that *J. communis* extracts exert antiproliferative effects in multiple experimental cancer models, including colorectal carcinoma (HCT116 and Caco-2) and pancreatic adenocarcinoma (PANC-1) cells, through mechanisms involving reduced cell viability and apoptosis induction [10,38,40]. Reported IC_50_ values vary depending on extraction solvent and plant origin, generally ranging between 80 and 250 µg/mL for crude extracts [41,42]. The aqueous extract of *J. communis* cone berries has been shown to induce cell death and enhance chemosensitivity in lung carcinoma (A549), hepatocellular carcinoma (HepG2), and prostate cancer (22RV1, DU145) cell lines through modulation of the p53 and PI3K/Akt signaling pathways [43].

Promising effects of an aqueous extract from *J. communis* cone berries on A375 melanoma cells were pointed out in our previous research [44]. Favorable anti-melanoma effects were also observed for other juniper species: In a study on *Juniperus turbinata* (syn. *J. phoenicea* var. *turbinata*), the essential oil produced a concentration-dependent antiproliferative effect (MTT, 72 h) and was reported as most active on A375 among the tested tumor lines, with an IC_50_ = 9.48 µg/mL (vs. 25.10 µg/mL on HCT116 and 33.69 µg/mL on MDA-MB-231). The antiproliferative activity of juniper-derived preparations has also been evaluated in other species. For comparison, an *n*-butanol fraction obtained from *Juniperus oxycedrus* berries was previously evaluated against a panel of human cancer cell lines, including A375 melanoma cells. The authors reported no significant reduction in A375 cell viability following treatment with the extract, whereas a measurable antiproliferative effect was observed in MCF-7 breast cancer cells. These findings suggest that the cytotoxic response elicited by *Juniperus*-derived extracts may be strongly influenced by factors such as species-specific phytochemical composition, extraction procedure, and the biological characteristics of the target cell line [45].

Among cutaneous malignancies, melanoma is characterized by a particularly aggressive clinical course and a strong propensity for metastatic dissemination. Despite representing a smaller proportion of skin cancer cases, it accounts for a substantial burden of disease and mortality worldwide. According to data reported by the Global Cancer Observatory [46], an estimated 331,722 new melanoma diagnoses and 58,667 melanoma-related deaths occurred globally in 2022, placing melanoma among the twenty most frequently diagnosed cancers worldwide. The age-standardized global incidence rate was estimated at approximately 3.2–4.2 cases per 100,000 individuals, with marked geographic variability, with the highest incidence being reported in Oceania, followed by North America and Europe [47]. Epidemiological analyses indicate that the global melanoma burden continues to rise and may reach nearly 510,000 new cases and 96,000 deaths annually by 2040 if current trends persist [48,49].

Current treatments for melanoma remain unsatisfactory, highlighting the need to explore new therapeutic approaches for this disease.

Considering the favorable results we previously obtained with an aqueous fraction of *J. communis* cone berries, as well as the positive findings reported for other species of the genus, we aimed to investigate additional extracts derived from the pseudo-fruits with respect to their cytotoxic effects on melanoma cells. Therefore, the present study aimed to evaluate ethyl acetate- and *n*-butanol-soluble fractions obtained from *J. communis* pseudo-fruits, with a particular focus on polyphenolic compounds of medium and higher polarity and their associated antioxidant, antimicrobial, and selective antimelanoma effects.

## 2. Materials and Methods

### 2.1. Extraction Protocol

Juniper pseudo-fruits were acquired from Stef Mar (Râmnicu Vâlcea, Romania) and authenticated at the Department of Pharmaceutical Botany, Faculty of Pharmacy, “Victor Babes” University of Medicine and Pharmacy Timisoara. A voucher sample of the cone berries was deposited in the Herbarium of the above-mentioned faculty (nr. JAA-01-23).

The extraction process was performed according to the following protocol: Three hundred grams of juniper pseudo-fruits was first mechanically crushed using an IKA A11 grinder (IKA-Werke GmbH & Co. KG, Staufen, Baden-Württemberg, Germany). The obtained plant material was then macerated in 1 L of 99.5% absolute ethanol (Riedel-de Haën, Fisher Scientific, Helsinki, Finland) at room temperature (22 ± 2 °C) for 24 h. Mechanical crushing reduced particle size and improved solvent penetration into the plant matrix, thereby enhancing the extraction efficiency of bioactive compounds. Following grinding, the plant material was kept immersed in absolute ethanol overnight to promote the diffusion of biologically active constituents from the pseudo-fruits into the solvent. To further enhance extraction efficiency, the mixture was subjected to ultrasonic treatment for 20 min using an ultrasonic bath (ELMA S120 Elmasonic, Elma Schmidbauer GmbH, Singen, Germany), which utilizes high-frequency sound waves to accelerate the extraction process.

Following ultrasonic treatment, the liquid phase containing the extracted constituents was separated from the residual plant material by filtration through Whatman Grade 4 filter paper (Cytiva, Maidstone, Kent, UK). The resulting filtrate was subsequently concentrated under reduced pressure using a rotary evaporator (Laborata 4000eco, Heidolph Instruments GmbH & Co. KG, Schwabach, Germany) to remove the extraction solvent. Ethanol was evaporated at 35 °C under reduced pressure to obtain a concentrated extract while preserving the integrity of the recovered bioactive constituent.

Upon solvent removal, a concentrated viscous extract containing the chemical constituents of juniper cone berries was obtained. The remaining plant residue was subjected to an additional extraction using the same procedure with a further 2 L of absolute ethanol. Overall, 300 g of plant material yielded 56.69 g of crude extract.

**Figure 1:**
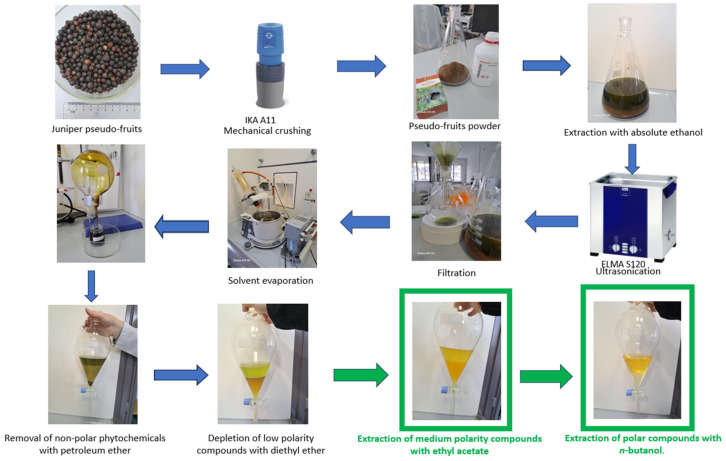
Workflow for the preparation of the ethyl acetate- and *n*-butanol-soluble fractions from *J. communis* pseudo-fruits.

Subsequently, the crude extract obtained from the juniper pseudo-fruits underwent a fractionation process. The crude extract (20 g) was reconstituted in 100 mL of distilled water, and the resulting suspension was placed in a separatory funnel for subsequent liquid–liquid partitioning. Liquid–liquid partitioning was then performed using organic solvents with increasing polarity. As our scope was to investigate fractions enriched in polyphenols of medium polarity, we initially removed non-polar phytochemicals through partitioning with petroleum ether, and subsequently depleted low-polarity compounds with diethyl ether. Next, we proceeded to partitions with ethyl acetate and *n*-butanol, obtaining our two fractions of interest, as these two solvents are known to efficiently extract polyphenols with medium and high polarity [50]. Each solvent extraction step was carried out three times using 150 mL of solvent per partition. The collected organic phases were evaporated separately to remove the solvents, yielding fractions with different polarity profiles (Figure 1). The present research focused on the ethyl acetate- and *n*-butanol-soluble fractions.

### 2.2. Phytochemical Analysis by HPLC-MS

#### 2.2.1. Qualitative Analysis

The qualitative profiling of phenolic compounds was performed using a Shimadzu Nexera X3 HPLC system (Shimadzu Corporation, Kyoto, Japan) coupled to a Shimadzu QTOF 9030 high-resolution mass spectrometer. The LC system was equipped with two LC-40D X3 pumps, an in-line degasser, a SIL-40C X3 autosampler, and a CTO-40S column oven. Chromatographic analyses were performed using a Zorbax SB-C18 reversed-phase column (100 × 3.0 mm, 3.5 μm; Agilent Technologies, Santa Clara, CA, USA), with the column temperature maintained at 48 °C.

The chromatographic system employed a binary mobile phase composed of acidified water (0.2% acetic acid, *v*/*v*; solvent A) and acidified methanol (0.2% acetic acid, *v*/*v*; solvent B). Separation was achieved using a gradient program increasing solvent B from 3% to 70% over 30 min, followed by a 4 min equilibration period at the initial conditions (3% B). The flow rate was maintained at 1 mL/min, and sample injections were performed with a volume of 1 μL. Mass spectrometric detection was carried out in negative electrospray ionization mode using high-resolution full-scan acquisition combined with an auto-MS/MS function. The ion source was operated under the following conditions: capillary voltage, −2000 V; interface temperature, 300 °C; nebulizing gas flow, 3 L/min; heating gas flow, 15 L/min; drying gas flow, 15 L/min; desolvation line temperature, 300 °C; and heat block temperature, 400 °C. The compounds were identified by spectral library match of MS/MS spectra obtained during analysis.

#### 2.2.2. Quantitative Analysis

For the quantitative determination, an Agilent 1100 Series HPLC system was used and it was coupled to an LC/MSD Ion Trap SL mass spectrometer (Agilent Technologies, Santa Clara, CA, USA). Chromatographic separations were performed using the same analytical column (Zorbax SB-C18, 100 mm × 3.0 mm i.d., 3.5 μm) and oven temperature (48 °C) described for the qualitative analysis. Two distinct, previously validated analytical methods were employed based on the target analytes [51,52,53]. Prior to HPLC analysis, the dry sub-extracts (ethyl acetate-soluble and *n*-butanol-soluble fractions) were solubilized in methanol, at a concentration of 1 mg/mL.

##### UV-Based Quantification

For quantitative analysis, chromatographic separation was performed using a binary solvent system comprising aqueous acetic acid (0.1%, *v*/*v*; solvent A) and methanol (solvent B). The mobile phase was delivered at 1 mL/min, and 5 μL of sample was injected for each analysis. The gradient program was initiated at 5% B, followed by a linear increase to 42% B over 35 min. This composition was maintained for an additional 3 min before returning to the initial conditions, with a 7 min equilibration step at 5% B [51,52]. Compound identification was supported by tandem mass spectrometry operating in negative electrospray ionization mode. The MS/MS parameters were set as follows: capillary voltage, +3000 V; nebulizer pressure, 60 psi; nitrogen drying gas flow, 12 L/min; and drying gas temperature, 360 °C. Quantitative determination of the selected analytes was based on UV detection, with the wavelength programmed at 330 nm during the first 17 min of the chromatographic run and at 370 nm from 17 to 38 min [51].

##### MS-Based Quantification

Quantification of an additional set of phenolic acids and catechins was carried out using a second validated analytical protocol. Chromatographic separation was achieved under modified gradient conditions, beginning with 3% solvent B and gradually increasing to 8% within the first 3 min, followed by a further increase to 20% B over the subsequent 5.5 min. The mobile-phase composition was then kept constant until the 10th minute, after which the initial chromatographic conditions were restored to allow column stabilization. A 5 μL injection volume was used throughout the analysis. For these compounds, analyte quantification relied on mass spectrometric detection, while compound identification was established through comparison of the recorded MS/MS fragmentation patterns with those of the corresponding reference standards [51,53].

##### Data Analysis

Compound identification was achieved by comparing the obtained mass spectra and chromatographic traces with those of reference standards from spectral libraries. The first analytical method relied on MS for positive structural confirmation, followed by UV-based quantification using analytical standard calibration curves, whereas the second analytical method utilized MS for both structural identification and quantification. Data processing was carried out using DataAnalysis (v5.3) and ChemStation (vB01.03) software (Agilent Technologies), and the concentrations of the identified compounds were expressed as μg/mL of plant extract.

### 2.3. Total Polyphenol Content

The total phenolic content was quantified using the Folin-Ciocâlteu (FC) assay, following a modified protocol described by Conforti et al. [54]. For total phenolic content determination, the extract was mixed with an acetone–methanol–water solvent system acidified with acetic acid and incubated at 60 °C for 1 h. After cooling, the assay was performed in triplicate using sodium carbonate as the alkaline reagent. Absorbance was recorded at 726 nm with an Agilent BioTek Synergy H1 Hybrid Multi-Mode Reader, and results were expressed as mg chlorogenic acid equivalents per gram of extract (mg CAE/g extract).

### 2.4. FTIR Spectroscopy

The functional group composition of the phytochemicals present in the ethyl acetate and *n*-butanol fractions of *J. communis* was investigated by Fourier transform infrared (FTIR) spectroscopy. Spectra were recorded using a Prestige-21 FTIR instrument (Shimadzu, Duisburg, Germany). Spectral interpretation was performed by matching the absorption bands recorded for the analyzed fractions with reference spectra of previously characterized compounds available in the literature and spectral databases [55]. FTIR spectra were acquired at room temperature using KBr pellets in the 4000–400 cm^−1^ range at a resolution of 4 cm^−1^.

### 2.5. Antioxidant Activity

The antioxidant potential of the investigated fractions was assessed using the DPPH (2,2-diphenyl-1-picrylhydrazyl) radical scavenging assay according to a protocol previously established by our research group, while the complete experimental workflow was described in detail in our earlier study on *Juniperi galbulus* [44]. Briefly, DPPH reagent (Sigma-Aldrich, Steinheim, Germany) was prepared as a 0.1 mM solution in 95% ethanol (*v*/*v*) and maintained under dark conditions at 4 °C until use. Ascorbic acid (Lach-Ner, Prague, Czech Republic) was employed as a reference antioxidant and prepared at a concentration of 1 mM in 95% ethanol. The ethyl acetate and *n*-butanol fractions were tested at concentrations of 0.1, 0.3, 0.5, 0.8, and 1 mg/mL. Antioxidant activity was evaluated spectrophotometrically at 517 nm using a UviLine 9400 instrument (SI Analytics, Mainz, Germany) and monitored at 5-s intervals over a total period of 20 min. To characterize both the immediate and time-dependent antioxidant responses, absorbance values recorded at 5 s and 1200 s were selected as representative time points corresponding to the initial and final phases of the scavenging reaction [56]. Antioxidant activity (AOA%) was calculated using the following equation:

AOA (%) = [(A_control_ − A_sample_)/A_control_] × 100

where A_control_ represents the absorbance of the DPPH solution without tested extract, while A_sample_ corresponds to the absorbance measured in the presence of the investigated fractions.

### 2.6. Antimicrobial Activity

The antimicrobial potential of the investigated fractions was assessed against selected reference microbial strains according to standardized EUCAST and CLSI recommendations, using a protocol previously developed by our research group, while the detailed experimental methodology was described in our earlier study on *Juniperi galbulus* [44]. The following reference strains (ThermoScientific, Waltham, MA, USA) were included: *Staphylococcus aureus* ATCC 25923, *Streptococcus pyogenes* ATCC 19615, *Escherichia coli* ATCC 25922, *Pseudomonas aeruginosa* ATCC 27853, and *Candida parapsilosis* ATCC 22019.

Reference bacterial strains were cultured on Columbia agar supplemented with 5% sheep blood, whereas *Candida parapsilosis* was maintained on Sabouraud agar containing chloramphenicol (Oxoid, Wesel, Germany). Microbial inocula were prepared in sterile 0.85% saline solution and adjusted to a turbidity equivalent to a 0.5 McFarland standard, corresponding to approximately 1–2 × 10^8^ CFU/mL.

Antimicrobial activity was initially evaluated using the disk diffusion assay. Mueller–Hinton agar was employed for most bacterial strains, while *Streptococcus pyogenes* was cultivated on Mueller–Hinton agar supplemented with sheep blood and β-NAD (MHF; Oxoid, Wesel, Germany). Sterile paper disks (6 mm diameter; BioMaxima, Lublin, Poland) impregnated with 5 μL of extract solution (20 mg/mL) were placed onto the surface of the inoculated agar plates.

Appropriate positive controls were represented by gentamicin or fluconazole, depending on the tested microbial strain, while sterile distilled water served as the control treatment. Following incubation at 35 °C for 24 h, antimicrobial activity was assessed by measuring the diameter of the inhibition zones surrounding the disks. Inhibition zones below 15 mm were interpreted as indicating low susceptibility under the applied screening conditions, without implying clinical resistance. The 15 mm inhibition diameter was used only as an arbitrary screening cut-off for selecting samples for subsequent MIC determination. Samples exhibiting inhibition zones larger than 15 mm were further subjected to minimum inhibitory concentration (MIC) determination according to the protocol described previously [44].

### 2.7. Cell-Based Assays

#### 2.7.1. Cell Culture

The human immortalized keratinocyte cell line HaCaT (catalogue no. 300493) was obtained from CLS Cell Lines Service GmbH (Eppelheim, Germany), whereas A375 human melanoma cells (ATCC^®^ CRL-1619™) were acquired from the American Type Culture Collection (Manassas, VA, USA). Both cell lines were maintained in Dulbecco’s Modified Eagle Medium (DMEM) supplemented with 10% fetal bovine serum and 1% penicillin–streptomycin solution (10,000 U/mL). Cell cultures were incubated at 37 °C under humidified conditions in an atmosphere containing 5% CO_2_.

#### 2.7.2. Cell Viability Evaluation

The effects of the investigated extracts on cell viability were evaluated in HaCaT keratinocytes and A375 melanoma cells using the Alamar Blue assay after 24 h of treatment. Cells were plated at a density of 1 × 10^4^ cells/well in 96-well culture plates and maintained until adequate attachment was achieved. Thereafter, the growth medium was replaced with treatment medium containing *J. communis* extracts at concentrations ranging from 25 to 300 μg/mL. The extracts were solubilized in DMSO; the final concentration of DMSO used in the control and experimental wells was 0.5% (*v*/*v*), a concentration at which cell viability was not influenced. After the treatment period, 10 µL/well of Alamar Blue solution (0.015% *w*/*v*) was added, and the plates were incubated for an additional 3 h to allow development of the fluorescent signal. This incubation time was selected according to the established assay protocol and provided an adequate fluorescence signal for viability assessment. The fluorescence signal was recorded using a Synergy HTX Multi-Mode Reader microplate reader (BioTek Instruments, Winooski, VT, USA), using excitation wavelengths of 528 nm and emission of 590 nm. Each experiment was performed in triplicate.

#### 2.7.3. Immunofluorescence Assay

Cells were treated for 24 h with *J. communis* extracts (ethyl acetate and *n*-butanol fractions, 100 μg/mL), fixed with 4% paraformaldehyde for 15 min, permeabilized with 0.1% Triton X-100 for 15 min, and blocked with 3% BSA for 30 min at room temperature. The actin cytoskeleton was visualized by immunofluorescence using a mouse monoclonal anti-β-actin antibody (Product # MA1-140, Thermo Fisher Scientific, Inc., Waltham, MA, USA; 1:2000 dilution), followed by incubation with an Alexa Fluor Plus 488-conjugated goat anti-mouse IgG (H+L) secondary antibody (Product # A32723, Thermo Fisher Scientific, Inc., Waltham, MA, USA; 1:500 dilution) for at least 30 min in the dark. Nuclear counterstaining was performed with Hoechst 33342 (Product # 62249, Thermo Fisher Scientific, Inc., Waltham, MA, USA). Fluorescence micrographs were acquired using a Thermo Scientific EVOS™ M5000 Imaging System equipped with a 40× objective.

### 2.8. Statistical Analysis

Statistical analysis was performed using one-way ANOVA followed by Dunnett’s post hoc test for multiple comparisons of treated groups against the corresponding control group. Due to the limited number of experimental replicates, formal normality testing was not considered sufficiently robust; therefore, parametric analysis was applied based on the experimental design and inspection of data distribution and variance across groups. Differences were considered statistically significant at * *p* < 0.05, ** *p* < 0.01, and *** *p* < 0.001.

## 3. Results

### 3.1. HPLC-MS Analysis of Polyphenolic Phytochemicals

#### 3.1.1. Qualitative Analysis

The qualitative LC-MS/MS profiling identified a total of 25 distinct phenolic compounds, consisting of a variety of phenolic acids, flavonoids, and their corresponding glycosylated derivatives, within the *J. communis* extracts (Table 1). A comparative evaluation of the phytochemical composition revealed significant differences in complexity between the two solvent fractions. The ethyl acetate fraction displayed a more diverse polyphenolic profile, successfully retaining 25 compounds. This comprehensive recovery indicates that ethyl acetate acts as a highly efficient solvent for isolating a broad spectrum of these secondary metabolites, comprising both aglycones and glycosides. In contrast, the *n*-butanol fraction demonstrated a notably narrower selectivity, retaining only 10 of the 25 identified compounds. While the *n*-butanolic partitioning successfully recovered certain flavonoids, namely rutin, hyperoside, and apigenin, along with specific glycosylated derivatives, it was entirely devoid of several phenolic acids, including gallic, chlorogenic, and *p*-coumaric acids. These low-molecular-weight phenolic acids, alongside other specific flavonoid derivatives, partitioned exclusively into the ethyl acetate phase. Therefore, the qualitative data demonstrates that the ethyl acetate fraction is richer in phenolic diversity, making it a more phytochemically complex extract compared to the *n*-butanol fraction.

#### 3.1.2. Quantitative Analysis

The quantitative profiling of polyphenolic constituents in *J. communis* fractionated extracts revealed a distinct distribution pattern dependent upon the solvent polarity. The LC-MS analysis demonstrated that hyperoside was the predominant compound, being consistently identified and quantified in both fractions, with a significantly higher concentration in the ethyl acetate fraction (3.726 ± 0.112 μg/mL) compared to the *n*-butanol fraction (0.824 ± 0.041 μg/mL). This finding indicates a preferential affinity of this flavonoid glycoside for moderately polar solvents and suggests its major contribution to the phytochemical profile of the ethyl acetate extract.

Furthermore, the analysis highlighted solvent-specific partitioning behaviors among the minor constituents. A selective distribution was observed for rutoside, which was quantified exclusively in the *n*-butanol fraction (0.281 ± 0.019 μg/mL) while remaining below the limit of quantification (<LOQ) in the ethyl acetate phase. This highlights the tendency of more polar flavonoid glycosides to partition into higher polarity solvents. In contrast, apigenin, a less polar flavonoid aglycone, was quantified only in the ethyl acetate fraction (0.192 ± 0.013 μg/mL) and was not detected in the *n*-butanol extract, further supporting the influence of solvent polarity on compound recovery.

The second quantitative LC-MS method employed for the quantification of additional phenolic acids and catechins revealed further differences in the chemical composition of tested extracts. Notably, solely protocatechuic acid was quantified, and it was detected exclusively in the ethyl acetate fraction (0.244 ± 0.036 μg/mL) (Table 2).

### 3.2. Total Polyphenol Content (TPC)

Determination of total phenolic content using the FC method demonstrated that solvent fractionation effectively concentrated phenolic constituents in the semi-polar fractions obtained from *J. communis* pseudo-fruits. This finding indicates a preferential distribution of these compounds according to solvent polarity. Specifically, the ethyl acetate fraction exhibited a high total polyphenolic content of 361.08 ± 17.72 mg chlorogenic acid equivalents (CAE)/g extract, whereas the *n*-butanol fraction contained 104.73 ± 10.03 mg CAE/g extract, indicating a preferential partitioning of phenolic compounds into solvents of intermediate polarity.

These values are considerably higher than those reported for several medicinal plant extracts quantified in CAE, such as *Teucrium montanum* (59.8 mg CAE/g extract) [57], *Cynara scolymus* (17.74 mg CAE/g extract), *Taraxacum officinale* (77.6 mg CAE/g extract), and *Cichorium intybus* (77.0 mg CAE/g extract) [58], highlighting the strong phenolic enrichment achieved through solvent fractionation. The substantially elevated polyphenolic content of the ethyl acetate fraction suggests a high concentration of flavonoid glycosides and other moderately polar phenolics, which are known contributors to antioxidant activity.

### 3.3. FTIR Spectroscopy

**Figure 2:**
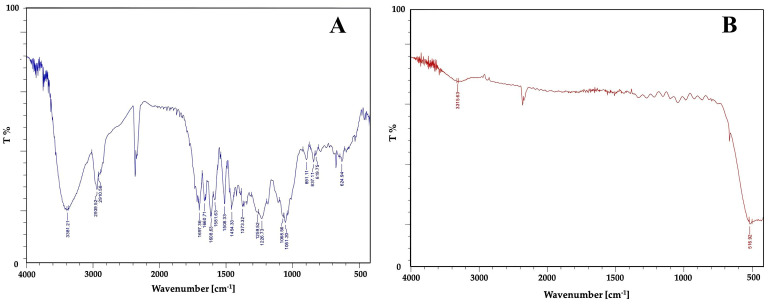
FTIR spectrum of *J. communis* extract (ethyl acetate fraction) (**A**) and FTIR spectrum of *J. communis* extract (*n*-butanol fraction) (**B**).

The FTIR spectrum of the *J. communis* ethyl acetate fraction revealed absorption bands consistent with the presence of phenolic constituents, including flavonoid derivatives. The broad absorption centered at 3381.2 cm^−1^ is characteristic of O–H stretching vibrations associated with hydroxyl groups commonly found in polyphenolic structures. Signals detected in the 2939.5–2910.5 cm^−1^ region indicate aliphatic C–H stretching vibrations originating from methyl and methylene groups. The intense absorption observed at 1697.3 cm^−1^ suggests the occurrence of conjugated carbonyl functionalities, while the spectral region between 1660.7 and 1581.6 cm^−1^ is consistent with aromatic and conjugated unsaturated structures. Additional absorptions at 1508.3 and 1454.3 cm^−1^ further support the presence of substituted aromatic rings, whereas the signal at 1373.3 cm^−1^ may be attributed to deformation vibrations of C–H bonds and phenolic hydroxyl groups. Overall, the spectral profile corroborates the enrichment of the ethyl acetate fraction in phenolic constituents, in agreement with the LC-MS/MS characterization and total polyphenol content results (Figure 2A).

In the case of *J. communis n*-butanol fraction, the FTIR spectrum revealed the presence of few absorption bands corresponding to hydroxylated, aliphatic, and low-frequency fingerprint vibrations. The broad absorption band at 3315.6 cm^−1^ can be assigned to O–H stretching vibrations, indicating the presence of hydroxyl-containing compounds, such as alcohols or phenols. A narrow absorption band around 2350 cm^−1^ is most likely related to atmospheric CO_2_ and should be interpreted with caution as an artifact rather than a structural feature. The very strong absorption in the low-wavenumber region, with a marked band at 516.9 cm^−1^, may be associated with fingerprint-region vibrations such as C-X stretching (Figure 2B).

### 3.4. Antioxidant Activity

Among the two tested extracts, the ethyl-acetate soluble fraction displayed consistently a higher antioxidant activity in the DPPH assay than did the *n*-butanol-soluble fraction (Table 3). Both extracts exhibited a concentration-dependent antioxidant activity. Higher concentrations of the ethyl acetate fraction, particularly 0.8 and 1.0 mg/mL, displayed antioxidant activities comparable to that of the reference compound ascorbic acid tested at 0.0176 mg/mL, corresponding to 0.1 mM. The *J. communis n*-butanol fraction showed a significantly lower radical scavenging capacity.

The *J. communis* ethyl acetate fraction showed a marked increase in antioxidant activity with increasing concentration, from 49.29 ± 0.04% at 0.1 mg/mL to 88.53 ± 0.01% at 1 mg/mL, indicating a strong dose-dependent effect and suggesting the presence of antioxidant constituents with high free-radical scavenging potential. In contrast, the *J. communis n*-butanol fraction displayed only weak activity, with final values ranging from 28.22 ± 0.03% to 33.51 ± 0.04%, and a limited increase with concentration, indicating a much lower contribution of antioxidant compounds in this fraction. The increase in antioxidant activity (AOA) values between 5 and 1200 s for both fractions indicates that DPPH free radicals were progressively consumed during the reaction time, the effect being much more pronounced for the ethyl acetate fraction. The superior antioxidant activity of the *J. communis* ethyl acetate fraction may be correlated with a higher content of phenolic or other redox-active constituents extracted in ethyl acetate, while the lower activity of *J. communis n*-butanol may reflect a reduced concentration or lower reactivity of such compounds in the *n*-butanol fraction.

### 3.5. Antimicrobial Activity

The antimicrobial potential of the *J. communis* fractions was assessed by agar disk diffusion, and the resulting inhibition zone diameters are summarized in Table 4. Among the tested microbial strains, *Streptococcus pyogenes* was the most susceptible to the investigated fractions. However, only the ethyl acetate fraction produced an inhibition zone above the predefined 15 mm screening threshold, with an inhibition diameter of 17 mm and an MIC value of 10 mg/mL. These results indicate a limited-to-moderate antibacterial effect under the present in vitro screening conditions. The remaining tested microorganisms showed only low susceptibility, with inhibition zones below the threshold selected for further MIC determination.

In the cases of *Staphylococcus aureus* and *Candida parapsilosis*, a moderate susceptibility was recorded, with inhibition zones of 10 mm for both fractions. *Escherichia coli* showed lower sensitivity, with inhibition zones of 10 mm, and 8 mm for the ethyl acetate fraction, and *J. communis n*-butanol fraction, respectively. *Pseudomonas aeruginosa* proved to be the least sensitive bacterium.

### 3.6. Effect on Cell Viability

The ethyl acetate fraction of *J. communis* exhibited a dose-dependent cytotoxic effect on both HaCaT and A375 cell lines. In HaCaT cells, viability progressively decreased from 77.43 ± 3% at 50 μg/mL to 27.94 ± 1.8% at 300 μg/mL. The calculated IC_50_ value for this fraction was 160.73 μg/mL, indicating a moderate cytotoxic effect on normal keratinocytes.

In A375 melanoma cells, the ethyl acetate fraction demonstrated a more pronounced antiproliferative activity, with cell viability decreasing to 32.54 ± 7.1% at 200 μg/mL and 16.84 ± 7.2% at 300 μg/mL. The IC_50_ value was 115.01 μg/mL, suggesting an enhanced sensitivity of tumor cells compared to normal cells and indicating a degree of selective cytotoxicity.

The *n*-butanol fraction displayed a biphasic effect on HaCaT cells. At low concentrations (25–50 μg/mL), a slight, non-significant increase in viability was observed (up to ~117%), while at higher concentrations a cytotoxic effect became evident, reducing viability to 43.35 ± 8.3% at 300 μg/mL. The IC_50_ value for HaCaT cells was 274.18 μg/mL, indicating relatively low toxicity toward normal cells.

**Figure 3:**
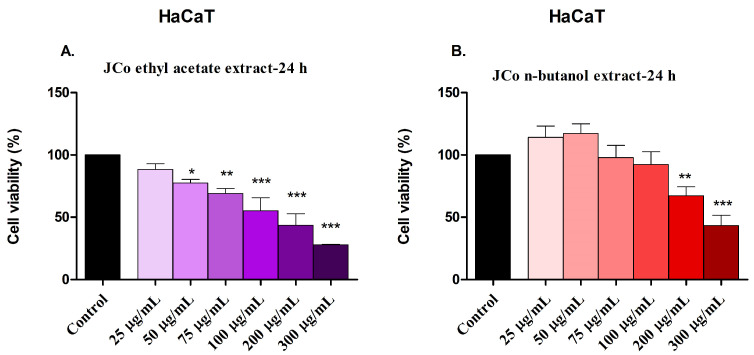
Cell viability of HaCaT cells after 24 h treatment with 25, 50, 75, 100, 200, and 300 μg/mL *J. communis* ethyl acetate fraction (**A**) and *n*-butanol fraction (**B**). Results are expressed as viability percentages normalized to vehicle-treated control cells, considered 100%. Control cells were maintained in culture medium containing the corresponding final concentration of DMSO used for extract solubilization (0.5% *v*/*v*). Data are presented as mean ± SD of three independent experiments performed in triplicate (* *p* < 0.05, ** *p* < 0.01, and *** *p* < 0.001 vs. vehicle control).

**Figure 4:**
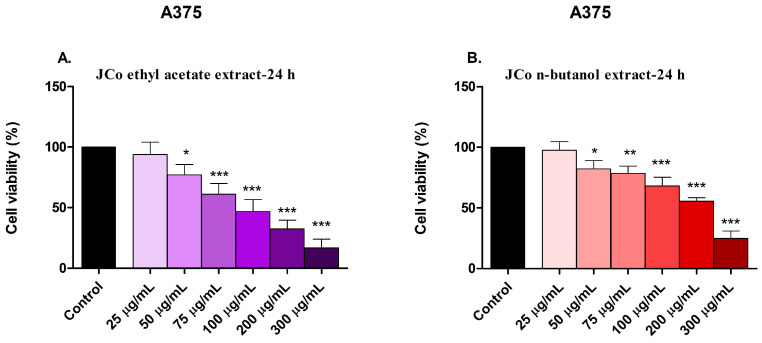
Cell viability of A375 cells after 24 h treatment with 25, 50, 75, 100, 200, and 300 μg/mL *J. communis* ethyl acetate fraction (**A**) and *n*-butanol fraction (**B**). Results are expressed as viability percentages normalized to vehicle-treated control cells, considered 100%. Control cells were maintained in culture medium containing the corresponding final concentration of DMSO used for extract solubilization (0.5% *v*/*v*). Data are presented as mean ± SD of three independent experiments performed in triplicate (* *p* < 0.05, ** *p* < 0.01, and *** *p* < 0.001 vs. vehicle control).

In contrast, the *n*-butanol fraction exerted a clear concentration-dependent inhibitory effect on A375 melanoma cells, with viability decreasing from 82.23 ± 6.7% at 50 μg/mL to 24.75 ± 6.2% at 300 μg/mL. The IC_50_ value was 189.85 μg/mL, reflecting a moderate cytotoxic effect, although less potent than that observed for the ethyl acetate fraction (Figure 3 and Figure 4).

### 3.7. Cell Morphology

In nonmalignant HaCaT cells, morphological changes varied depending on the extract. Treatment with the *J. communis* ethyl acetate fraction induced evident alterations at both 100 and 300 μg/mL, characterized by cell rounding, partial detachment, and a decrease in cell density, consistent with features of cellular stress and reduced viability.

**Figure 5:**
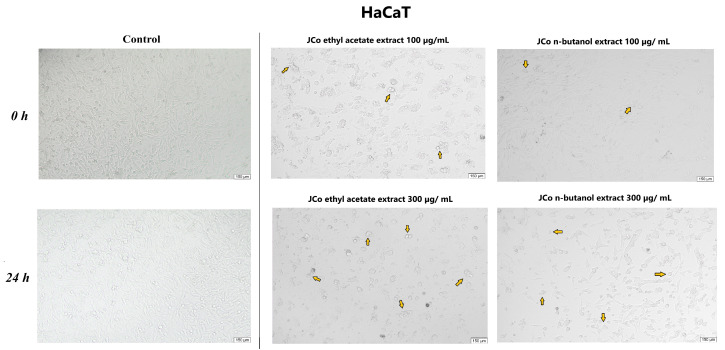
Evaluation of morphological changes in HaCaT cells after 24 h treatment with 100 and 300 μg/mL *J. communis* ethyl acetate and *n*-butanol fractions. Untreated cells served as control. The morphological alterations, including cell rounding, reduced confluence, and partial detachment, are indicated by yellow arrows. Images were acquired using an inverted microscope; scale bar: 150 μm.

In contrast, the *J. communis n*-butanol fraction produced minimal morphological changes at lower concentrations, with cells largely maintaining their normal epithelial appearance. Noticeable alterations, including cell rounding, detachment, and reduced confluence, were observed only at the highest concentration tested (300 μg/mL), indicating a weaker impact on cell morphology compared to the ethyl acetate fraction (Figure 5).

**Figure 6:**
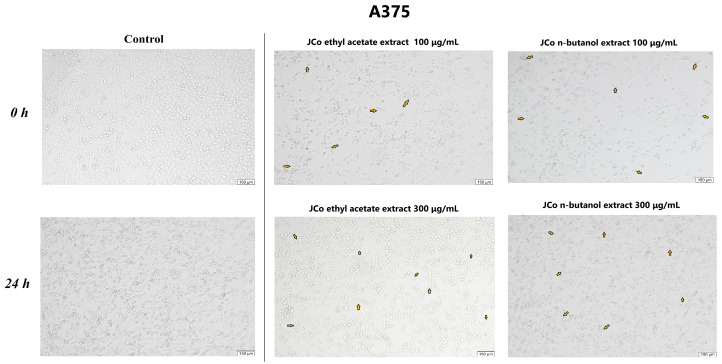
Evaluation of morphological changes in A375 melanoma cells after 24 h treatment with 100 and 300 μg/mL *J. communis* ethyl acetate and *n*-butanol fractions. Untreated cells served as control. The yellow arrows indicate treatment-related morphological alterations, including cell rounding, shrinkage, reduced confluence, loss of adhesion, and partial detachment. Images were acquired using an inverted microscope; scale bar: 150 μm.

In A375 melanoma cells, treatment with the *J. communis* ethyl acetate fraction at both tested concentrations (100 and 300 μg/mL) induced pronounced morphological alterations, including cell rounding, shrinkage, fragmentation, loss of adhesion, and detachment. These changes were accompanied by a marked reduction in cell density, consistent with the decreased viability observed in parallel assays. The *J. communis n*-butanol fraction also induced morphological changes in A375 cells, although to a lesser extent, characterized by cell rounding, partial detachment, and reduced confluence, particularly at higher concentrations (Figure 6).

**Figure 7:**
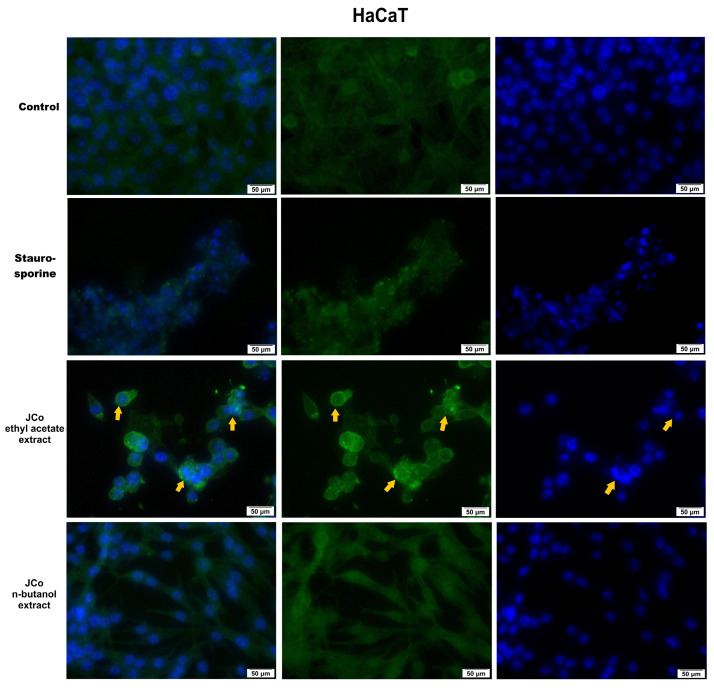
Effect of 24 h treatment with *J. communis* ethyl acetate and *n*-butanol fractions (100 μg/mL) on HaCaT cell nuclei (blue, Hoechst staining) and cytoskeleton (β-actin, green staining). Staurosporine (5 μM) was used as a positive control for apoptosis induction. Apoptosis-related morphological alterations, including nuclear shrinkage, chromatin condensation, nuclear fragmentation, and cytoskeletal reorganization, are indicated by yellow arrows. Images were acquired using an inverted microscope; scale bar: 150 μm.

Immunofluorescence analysis in HaCaT cells revealed that treatment with 100 μg/mL *J. communis n*-butanol fraction did not induce significant morphological alterations, with cells maintaining normal epithelial morphology, intact cytoskeletal organization, and nuclei of regular size and shape. In contrast, cells treated with the *J. communis* ethyl acetate fraction exhibited morphological features characteristic of apoptosis, including nuclear shrinkage, chromatin condensation, and fragmentation. These nuclear alterations were associated with pronounced cytoskeletal reorganization, changes in cell shape, and loss of structural integrity (Figure 7).

**Figure 8:**
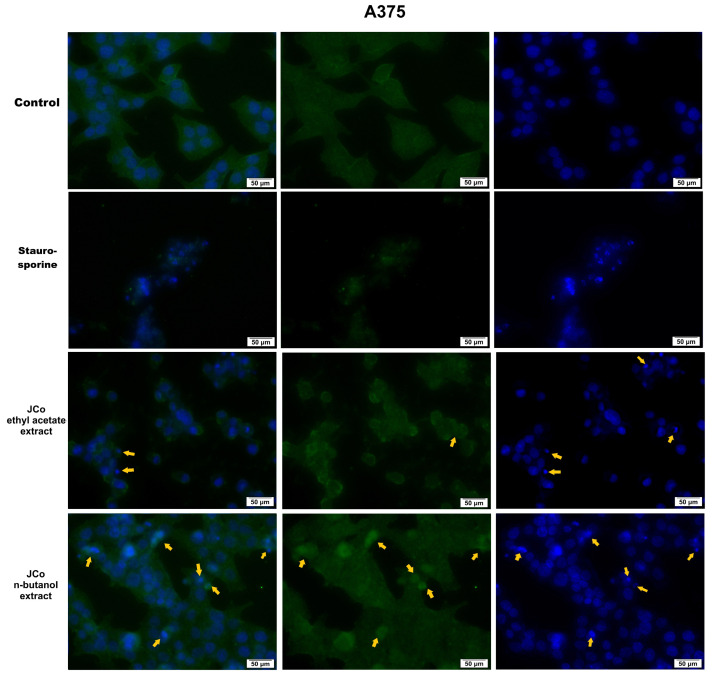
Effect of 24 h treatment with *J. communis* ethyl acetate and *n*-butanol fractions (100 μg/mL) on A375 cell nuclei (blue, Hoechst staining) and cytoskeleton (β-actin, green staining). Staurosporine (5 μM) was used as a positive control for apoptosis induction. The yellow arrows indicate apoptosis-related morphological alterations, including nuclear shrinkage, chromatin condensation, nuclear fragmentation, cytoskeletal reorganization, loss of structural integrity, and apoptotic body formation. Images were acquired using an inverted microscope; scale bar: 150 μm.

The immunofluorescence assay of A375 melanoma cells revealed that treatment with the *J. communis* ethyl acetate fraction (100 μg/mL) induced marked morphological alterations consistent with apoptotic cell death (Figure 8). Treated cells exhibited pronounced cytoskeletal reorganization, accompanied by changes in cell shape and loss of structural integrity. At the nuclear level, clear apoptotic features were observed, including chromatin condensation, nuclear shrinkage, and fragmentation. The presence of apoptotic bodies, appearing as small, dispersed, and variably sized condensed chromatin fragments, further supported the induction of apoptosis. In comparison, treatment with the *J. communis n*-butanol fraction (100 μg/mL) also induced morphological changes in A375 cells, although less pronounced. These included moderate cytoskeletal alterations, slight changes in cell shape, and partial loss of adhesion. Nuclear changes suggestive of early apoptosis, such as mild chromatin condensation and limited nuclear shrinkage, were observed, indicating a weaker pro-apoptotic effect relative to the ethyl acetate fraction.

## 4. Discussion

The biological effects observed in the present study may be associated with the extraction and fractionation procedures applied to the crude extract. Solvent partitioning is known to influence the distribution of phytochemicals according to their polarity, resulting in fractions with distinct chemical compositions. Consequently, differences in the phytochemical profile of the obtained fractions may contribute to the variations observed in their biological activities [17,24]. This concept is supported by the present findings, where the ethyl acetate fraction, enriched in medium-polarity flavonoids, exhibited superior antioxidant, antibacterial, and cytotoxic activities compared to the more polar *n*-butanol fraction.

### 4.1. Polyphenolic Profile

The HPLC analysis revealed that the investigated fractions have a notable content of phenolic compounds. The key compound is hyperoside (present in both fractions), while rutoside, protocatechuic acid, and apigenin are other characteristic phytochemicals (Table 1). The ethyl acetate fraction displayed a markedly higher content of hyperoside and exclusive detection of apigenin and protocatechuic acid, whereas the *n*-butanol fraction was enriched in rutoside. These findings are consistent with previous reports indicating that *J. communis* extracts contain flavonoids such as quercetin derivatives, whose distribution is strongly influenced by solvent polarity [17,24].

The preferential accumulation of hyperoside in the ethyl acetate fraction points to its enrichment in medium-polarity solvents, while the *n*-butanol fraction concentrates more polar compounds such as rutoside, a quercetin derivative bearing two sugar moieties. Similar fraction-dependent phytochemical distributions have been described in recent studies, confirming that solvent partitioning significantly impacts both qualitative and quantitative composition [25]. The biological activity of the investigated fractions may be partly attributed to the presence of phenolic constituents identified by LC-MS/MS analysis, including hyperoside, rutoside, apigenin, and protocatechuic acid. Previous studies have reported antioxidant, antiproliferative, and pro-apoptotic effects for these compounds in various experimental models. For example, hyperoside has been associated with the activation of mitochondria-dependent apoptotic pathways involving caspase signaling [59], while rutoside modulates oncogene expression and promotes apoptosis via regulation of key signaling cascades [60]. Apigenin exhibits broad-spectrum anticancer activity, including in melanoma models, where it significantly inhibits proliferation and induces apoptosis in A375 human melanoma cells through caspase activation and modulation of Akt/MAPK signaling pathways [32,61]. Similarly, protocatechuic acid exerts antitumoral effects in melanoma models by inhibiting tumor progression and metastasis. In B16F10 cells, it suppresses metastatic potential through downregulation of MMP-2 and inhibition of the Ras/Akt/NF-κB signaling pathway [62].

### 4.2. Polyphenol Content

Differences in total polyphenol content among the obtained fractions indicate that solvent partitioning substantially influenced the distribution of phenolic constituents in the *J. communis* pseudo-fruit extract. The ethyl acetate fraction exhibited a markedly higher total polyphenolic content (361.08 ± 17.72 mg CAE/g extract) compared to the *n*-butanol fraction (104.73 ± 10.03 mg CAE/g extract), demonstrating the efficiency of ethyl acetate as a solvent for the extraction of flavonoids. This behavior is consistent with the physicochemical properties of plant phenolics, which encompass a wide range of compounds with varying polarity. Flavonoids and phenolic acids of intermediate polarity are generally more efficiently extracted with solvents such as ethyl acetate, while highly polar constituents tend to remain in more polar fractions [63,64]. The observed enrichment of the ethyl acetate fraction therefore suggests an efficient concentration of redox-active phenolic compounds.

The markedly higher polyphenolic content observed in the ethyl acetate fraction suggests that selective solvent partitioning efficiently concentrates moderately polar phenolic constituents. However, direct quantitative comparisons with literature data should be interpreted cautiously, as total phenolic contents are frequently expressed using different calibration standards, including chlorogenic acid equivalents (CAE) and gallic acid equivalents (GAE), which may influence the reported absolute values [65,66].

The relatively elevated polyphenolic content observed in this study may be attributed to the applied fractionation strategy, which enhances the selectivity of extraction and allows the concentration of specific classes of bioactive compounds. Previous studies have demonstrated that extraction techniques and solvent polarity significantly influence the yield and composition of phenolic compounds in plant extracts [63,64]. In this context, solvent partitioning represents an effective approach for optimizing phytochemical profiles.

### 4.3. FTIR

FTIR analysis revealed spectral features consistent with the presence of phenolic constituents, including compounds bearing hydroxyl, carbonyl, and aromatic functionalities. The ethyl acetate fraction displayed a richer spectral profile, characterized by a greater number of absorption bands associated with conjugated and aromatic structures, whereas the *n*-butanol fraction exhibited fewer characteristic signals. These findings are consistent with the phytochemical characterization obtained by LC-MS/MS and the higher total polyphenol content determined for the ethyl acetate fraction.

The observed spectral characteristics are in line with previous reports describing Juniperus extracts as important sources of phenolic compounds and other aromatic phytochemicals [34]. The less complex spectral profile of the *n*-butanol fraction may reflect differences in its phytochemical composition relative to the ethyl acetate fraction.

### 4.4. Antioxidant Activity

Evaluation of free-radical scavenging activity revealed distinct antioxidant profiles for the *J. communis* fractions, highlighting the influence of solvent partitioning on the recovery of antioxidant constituents. The ethyl acetate fraction exhibited strong, concentration dependent radical scavenging activity, reaching 88.53 ± 0.01% at 1 mg/mL. In contrast, the *n*-butanol fraction showed consistently low activity, with final values not exceeding 33.51 ± 0.04%. These results indicate that antioxidant constituents of *J. communis* are preferentially extracted into solvents of intermediate polarity, such as ethyl acetate, rather than more polar solvents like *n*-butanol, in agreement with previous reports emphasizing the importance of solvent selection in maximizing antioxidant yield [17].

The antioxidant activity observed for the investigated fractions may be related to differences in their phenolic composition, as indicated by the HPLC and total polyphenol content analyses. Phenolic compounds, including flavonoids and phenolic acids identified in *J. communis* extracts, are known to contribute to radical scavenging activity through electron- and hydrogen-donating mechanisms. Previous studies have similarly reported variations in the antioxidant potential of juniper berry extracts, which have been attributed to differences in phytochemical composition associated with extraction procedures and plant origin [17,41,67]. In particular, ethyl acetate fractions have been shown to concentrate bioactive phenolic constituents with enhanced radical scavenging potential, which may explain the superior activity observed in the present study. The pronounced antioxidant activity observed for the ethyl acetate fraction supports the presence of compounds with high redox efficiency.

The progressive increase in antioxidant activity values between their assessment at 5 s, and 1200 s, respectively, supports the time-dependent interaction of phytochemicals with DPPH radicals, demonstrating a remarkable feature of plant-derived antioxidants: they associate both rapid initial scavenging effects [68] and sustained antioxidant action [69]. Such kinetic behavior is characteristic for phenolic-rich plant extracts and reflects the contribution of multiple compounds with different reactivity profiles [70]. While the rapid initial scavenging effects are needed for rapid cellular defense and prevention of early-stage oxidative damage, long-lasting, delayed effects maintain antioxidant activity and cellular protection over an extended period.

In contrast, the limited antioxidant activity of the *n*-butanol fraction may be attributed to its distinct phytochemical composition, likely enriched in highly polar constituents with lower radical scavenging efficiency. Previous studies have shown that polar fractions often contain compounds with reduced electron-donating capacity or steric limitations that hinder their interaction with DPPH radicals, resulting in weaker antioxidant responses [41]. This further supports the hypothesis that solvent polarity plays a significant role in selectively extracting compounds with high antioxidant potential.

### 4.5. Antimicrobial Activity

The investigated *J. communis* fractions exhibited measurable antimicrobial activity, although the magnitude of the response varied among the tested microorganisms. Differences in inhibitory effects were also observed between the fractions, suggesting an influence of their distinct phytochemical compositions. Although the ethyl acetate fraction demonstrated measurable activity against *Streptococcus pyogenes*, the obtained MIC value (10 mg/mL) indicates relatively weak antimicrobial potency according to commonly accepted criteria for plant-derived antimicrobial agents. *Staphylococcus aureus* was equally susceptible to both extracts. Conversely, Gram-negative bacteria such as *Escherichia coli* and *Pseudomonas aeruginosa* showed lower sensitivity. *Candida parapsilosis* displayed moderate susceptibility to both fractions.

This pattern is consistent with previously reported data on *Juniperus* species, where Gram-positive bacteria are generally more susceptible to plant-derived extracts than Gram-negative strains [24,66]. The increased sensitivity of Gram-positive bacteria may be attributed to the simpler structure of their cell wall, which lacks the outer membrane characteristic of Gram-negative bacteria. This outer membrane acts as an effective permeability barrier, limiting the penetration of hydrophobic and moderately polar bioactive compounds, including phenolics and terpenoids [24,71]. Comparable findings were reported for *J. communis* berry extracts, where antimicrobial activity varied according to phytochemical composition and extract type, with Gram-positive bacteria generally exhibiting greater susceptibility than Gram-negative microorganisms [67].

The greater susceptibility of *S. pyogenes* to the ethyl acetate fraction may be related to the enrichment of this fraction in phenolic constituents. These compounds have been reported to exert antimicrobial effects through multiple cellular targets, including membrane-associated structures and essential metabolic processes [27,66]. In contrast, the lower activity of the *n*-butanol fraction suggests a reduced content or lower bioavailability of such active constituents, as highly polar compounds often exhibit weaker interactions with microbial membranes [71].

The relatively low inhibition observed for *Pseudomonas aeruginosa* is in agreement with its well-documented intrinsic resistance to antimicrobial agents, which is largely due to its highly selective outer membrane, efflux pump systems, and biofilm-forming capacity [71]. Similarly, the moderate antifungal activity against *Candida parapsilosis* may reflect the ability of certain phytochemicals, particularly terpenoids and phenolic compounds, to interact with fungal cell membranes and alter their permeability, although the effect appears limited under the tested conditions [27].

Overall, the antimicrobial activity of the investigated fractions was limited under the present in vitro screening conditions. Only the ethyl acetate fraction tested against *Streptococcus pyogenes* produced an inhibition zone above the predefined 15 mm threshold, with an MIC value of 10 mg/mL. Although this result indicates a measurable antibacterial effect, the relatively high MIC suggests limited direct clinical relevance. The remaining tested microorganisms showed only low susceptibility, and no strong antimicrobial effect can be inferred from these data.

The slightly higher susceptibility of Gram-positive bacteria may be related to differences in cell wall architecture compared with Gram-negative bacteria, whose outer membrane acts as an important permeability barrier against hydrophobic and moderately polar compounds. However, these observations should be interpreted as preliminary screening data and require further validation using additional strains, lower active concentrations, and more detailed mechanistic assays.

### 4.6. Cytotoxic Activity

Melanoma represents the most aggressive form of skin cancer and is responsible for the majority of skin cancer-related mortality due to its high metastatic potential. Although early-stage melanoma is associated with a favorable prognosis, with five-year survival rates exceeding 99%, survival decreases significantly in advanced stages, reaching approximately 30–35% in metastatic disease [72]. Moreover, the global incidence of melanoma continues to rise, with substantial geographic variation largely attributed to differences in ultraviolet radiation exposure and population-related risk factors [73]. In this context, increasing attention has been directed toward plant-derived bioactive compounds as potential sources of complementary therapeutic agents with anticancer properties [74]. These findings further support the notion that the biological activity of *J. communis* extracts may vary considerably according to phytochemical composition and external factors affecting metabolite accumulation [41,42]. Previous studies have demonstrated that *Juniperus* extracts can inhibit tumor cell proliferation and induce apoptosis in cancer models, supporting the biological relevance of the present findings [75]. The enhanced activity of the ethyl acetate fraction observed in this study may be attributed to its enrichment in bioactive flavonoids and phenolic acids, whereas the *n*-butanol fraction likely contains more polar constituents with comparatively lower cytotoxic potency. Comparable antiproliferative effects have been previously reported for *Juniperus* extracts, where bioactive constituents were associated with apoptosis induction and inhibition of tumor cell proliferation [76]. From a mechanistic perspective, the cytotoxic effects observed may be associated with the presence of polyphenolic compounds identified in the analyzed fractions. Among these, apigenin is well documented in melanoma models, including A375 cells, where it reduces cell viability, inhibits migration, and induces apoptosis through activation of caspase-dependent pathways and modulation of ERK and Akt/mTOR signaling [61,77]. In addition, rutoside has been reported to enhance oxidative stress-mediated cytotoxicity and apoptosis in A375 melanoma cells, particularly in combination-based photodynamic experimental approaches involving reactive oxygen species accumulation [78]. Although protocatechuic acid is less frequently associated with direct cytotoxic effects in A375 cells, its anti-melanoma activity has been demonstrated in B16F10 models, where it inhibits metastatic progression by downregulating MMP-2 expression and suppressing Ras/Akt/NF-κB signaling pathways [62]. Hyperoside may also contribute to the observed antiproliferative effects, as previous studies have reported its ability to modulate apoptosis- and oxidative stress-related pathways in skin cancer models. However, its specific role in melanoma remains insufficiently characterized; therefore, its contribution should be interpreted cautiously and may reflect synergistic interactions with other polyphenolic constituents present in the investigated fraction [79].

Taken together, these data support the hypothesis that the cytotoxic effects observed in the present study may be associated with medium polarity polyphenols capable of modulating redox balance and apoptosis-related pathways in melanoma cells, thereby highlighting the potential of *J. communis* fractions as sources of bioactive compounds with anticancer relevance.

## 5. Conclusions

The present study demonstrated that solvent fractionation significantly influences the phytochemical profile and biological activity of *J. communis* pseudo-fruit extracts. The ethyl acetate fraction was more enriched in phenolic compounds and showed stronger antioxidant activity and higher antiproliferative effects against A375 melanoma cells than the *n*-butanol fraction, while displaying lower toxicity toward normal HaCaT keratinocytes. These effects were supported by treatment-related morphological and apoptosis-associated changes, suggesting that medium-polarity polyphenols may contribute to the selective antimelanoma activity observed.

The antimicrobial activity of both fractions was generally limited, with the most noticeable effect observed for the ethyl acetate fraction against *S. pyogenes*.

Overall, these findings indicate that *J. communis* fractions may represent a promising source of bioactive compounds with antioxidant and selective anticancer relevance. However, the present study is limited to in vitro investigations, and further studies are required to clarify the molecular mechanisms involved and to validate these findings in in vivo experimental models.

## Figures and Tables

**Table 1:** Results of the qualitative LC-MS/MS analysis.

No.	Compound Name	Molecular Formula	Retention Time (min)	Precursor Ion *m*/*z*	MS/MS Ion 1 *m*/*z*	MS/MS Ion 2 *m*/*z*	*J. communis* *n*-Butanol Fraction	*J. communis* Ethyl Acetate Fraction
1	Protocatechuic acid-4-O-glucoside	C_13_H_16_O_9_	2.7	315.072	153.019	109.029	yes	yes
2	3,4-Dihydroxyphenyllactic acid-O-glucoside	C_15_H_20_O_10_	3.34	359.098	197.045	179.034	no	yes
3	Vanillic acid-4-O-glucoside	C_14_H_18_O_9_	3.74	329.087	167.034	152.011	yes	yes
4	Protocatechuic aldehyde	C_7_H_6_O_3_	3.96	137.024	108.021	136.016	no	yes
5	Catechin-O-glucoside	C_21_H_24_O_11_	4.1	451.124	289.071	245.081	yes	yes
6	Aromadendrin-O-Glucoside	C_21_H_22_O_11_	8.6	449.108	287.056	259.061	yes	yes
7	Luteolin-7-O-glucoside	C_21_H_20_O_11_	15.04	447.093	285.04	133.029	no	yes
8	Quercetin-3-O-robinobioside	C_27_H_30_O_16_	15.16	609.146	301.035	151.003	yes	yes
9	Quercetin-3-O-arabinoside	C_20_H_18_O_11_	16	433.076	300.027	301.033	yes	yes
10	Kaempferol-3-O-galactoside	C_21_H_20_O_11_	16.71	447.093	284.032	285.04	yes	yes
11	Kaempferol-3-O-arabinoside	C_20_H_18_O_10_	17.7	417.081	284.032	255.029	no	yes
12	Gallic acid	C_7_H_6_O_5_	1.362	169.014	125.024	79.018	no	yes
13	Protocatechuic acid	C_7_H_6_O_4_	2.672	153.019	109.029	91.018	yes	yes
14	Gentisic acid	C_7_H_6_O_4_	3.30	153.019	109.029	108.021	no	yes
15	Catechin	C_15_H_14_O_6_	5.812	289.071	245.081	205.05	no	yes
16	Vanillic acid	C_8_H_8_O_4_	6.389	167.034	152.011	123.045	no	yes
17	Chlorogenic acid	C_16_H_18_O_9_	6.451	353.087	191.056	179.034	no	yes
18	*p*-Coumaric acid	C_9_H_8_O_3_	9.2	163.04	119.05	163.04	no	yes
19	Umbeliferone	C_9_H_6_O_3_	9.6	161.024	117.034	161.024	no	yes
20	Hyperoside	C_21_H_20_O_12_	14.9	463.088	300.027	301.035	yes	yes
21	Isoquercitrin	C_21_H_20_O_12_	15.35	463.088	300.027	301.035	no	yes
22	Rutoside	C_27_H_30_O_16_	15.5	609.146	301.035	300.027	yes	yes
23	Apigenin 7-glucoside	C_21_H_20_O_10_	17	431.098	269.045	117.034	yes	yes
24	Quercitrin	C_21_H_20_O_11_	17.11	447.093	301.035	300.027	no	yes
25	Apigenin	C_15_H_10_O_5_	23.25	269.045	117.034	151.003	no	yes

MS/MS ion 1 and MS/MS ion 2 are the two most abundant product ions from the mass spectra of parent ion.

**Table 2:** Identification and quantification of polyphenolic compounds from *J. communis* extracts by HPLC-MS.

Compound	Concentration (μg/mL)
***J. communis*** **ethyl acetate fraction**	
Protocatechuic acid	0.244 ± 0.036
Hyperoside	3.726 ± 0.112
Rutoside	<LOQ
Apigenin	0.192 ± 0.013
***J. communis n*** **-butanol fraction**	
Protocatechuic acid	ND
Hyperoside	0.824 ± 0.041
Rutoside	0.281 ± 0.019
Apigenin	ND

Values are expressed as mean ± SD (n = 3). <LOQ—detected below the limit of quantification; ND—not detected under the experimental conditions.

**Table 3:** The antioxidant activity (AOA) of *J. communis* ethyl acetate fraction and *n*-butanol fraction as compared with ascorbic acid.

Tested Sample	Concentration	Initial AOA [%] (at 5 s)	Final AOA [%] (at 1200 s)
Ascorbic acid	0.0176 mg/mL	84.98 ± 0.13	86.74 ± 0.02
*J. communis* ethyl acetate fraction	0.1 mg/mL	39.46 ± 1.42	49.29 ± 0.04
	0.3 mg/mL	58.88 ± 3.08	84.07 ± 0.01
	0.5 mg/mL	67.95 ± 2.59	85.66 ± 0.04
	0.8 mg/mL	80.99 ± 2.35	86.50 ± 0.03
	1.0 mg/mL	83.43 ± 1.88	88.53 ± 0.01
*J. communis* *n*-butanol fraction	0.1 mg/mL	26.88 ± 0.15	28.22 ± 0.03
	0.3 mg/mL	27.36 ± 0.04	29.51 ± 0.01
	0.5 mg/mL	27.57 ± 0.01	30.21 ± 0.01
	0.8 mg/mL	28.10 ± 0.11	32.48 ± 0.04
	1.0 mg/mL	28.38 ± 0.09	33.51 ± 0.04

The values of AOA% are expressed as mean ± SD (n = 3).

**Table 4:** Inhibition zone diameters (mm) of *J. communis* extracts against selected microbial strains determined by the disk diffusion method.

Microbial Strains	*J. communis* Fraction	Inhibition Zone (mm)
*Streptococcus pyogenes* ATCC 19615	ethyl acetate *n*-butanol	17 10
*Staphylococcus aureus* ATCC 25923	ethyl acetate *n*-butanol	10 10
*Escherichia coli* ATCC 25922	ethyl acetate *n*-butanol	10 8
*Pseudomonas aeruginosa* ATCC 27853	ethyl acetate *n*-butanol	8 8
*Candida parapsilosis* ATCC 22019	ethyl acetate *n*-butanol	10 10

## Data Availability

The original contributions presented in this study are included in the article. Further inquiries can be directed to the corresponding authors.
